# Understanding of the role of serum creatinine in a subset of the Brazilian population

**DOI:** 10.1590/2175-8239-JBN-2023-0117en

**Published:** 2024-01-22

**Authors:** Bruno Pellozo Cerqueira, Júlia Ferreira Rocha, Rafaela Francisquetti Barnes, Pedro Henrique Moretti Pepato, Thays Sellan Paim, Francisco De Nardi, Fabrício Akira Hsu, Juliana Miki Oguma, Leticia Miyuki Ito, Enio Yasuhiro Arimatsu Policarpo da Silva, Alexandre Vizzuso-Oliveira, Fernando Diniz dos Santos Filho, João Vitor Bozza Maia, Juan Diego Zambrano Mendez, Beatrice Borges Sato, Roberto Matias Souza, Andre Kiyoshi Miyahara, Gianna Mastroianni Kirsztajn

**Affiliations:** 1 Universidade Federal de São Paulo, Escola Paulista de Medicina, Departamento de Medicina, São Paulo, SP, Brazil.

**Keywords:** Creatinine, Health Education, Knowledge, Renal Insufficiency, Chronic, Creatinina, Educação em Saúde, Conhecimento, Insuficiência Renal Crônica

## Abstract

**Introduction:**

Chronic kidney disease is usually asymptomatic, and its diagnosis depends on laboratory tests, with emphasis on serum creatinine and proteinuria.

**Objective:**

To assess knowledge on the role of serum creatinine as a biomarker of kidney function in a sample of the Brazilian population.

**Method:**

Cross-sectional observational study conducted in São Paulo (SP, Brazil), in which a random adult population was interviewed.

**Results:**

A total of 1138 subjects were interviewed, with a median age of 36 years old (27–52); 55.1% were female. Regarding the “creatinine” biomarker, 40.6% stated they had never performed such a test. When asked about their knowledge on the usefulness of this exam, only 19.6% knew its function. The other responses were “I don’t know” (71.6%), evaluating heart function (0.9%) and liver function (7.8%). Of those who reported they had already taken a creatinine test, only 29.4% correctly identified the role of creatinine. When dividing the groups into “knows” and “does not know” the function of creatinine, a statistically significant difference (p < 0.05) was observed regarding level of education, female sex, being a healthcare student/worker, having ever measured creatinine, knowing someone with kidney disease and older age. In the multivariate analysis, the main variable related to knowing the creatinine role was having previously taken the test (OR 5.16; 95% CI 3.16–8.43, p < 0.001).

**Conclusion:**

There is a significant lack of knowledge about creatinine and its use in checkups. The results indicate that greater efforts are needed from healthcare professionals to raise awareness on the role of serum creatinine.

## Resumo

**Introdução:**

A doença renal crônica costuma ser assintomática e seu diagnóstico depende da realização de exames laboratoriais, com destaque para a creatinina sérica e pesquisa de proteinúria.

**Objetivo:**

Avaliar em uma amostra da população brasileira o conhecimento sobre o papel da creatinina sérica como marcador de função renal.

**Método:**

Estudo observacional transversal realizado na cidade de São Paulo (SP, Brasil), em que foi entrevistada uma população adulta aleatória.

**Resultados:**

Foram entrevistados 1138 indivíduos, com idade mediana de 36 anos (27–52); 55,1% do sexo feminino. Com relação ao marcador “creatinina”, 40,6% afirmaram que nunca realizaram tal dosagem. Quando questionados quanto ao conhecimento sobre a utilidade desse exame, somente 19,6% sabiam a sua função. As outras respostas foram “não sei” (71,6%), avaliar o funcionamento do coração (0,9%) e fígado (7,8%). Dos que afirmaram já terem realizado o exame de creatinina, somente 29,4% acertaram a função da creatinina. Ao dividir os grupos em “sabe” e “não sabe” a função da creatinina, percebeu-se diferença estatisticamente significante (p < 0,05) em relação ao grau de escolaridade, sexo feminino, ser aluno/trabalhador da saúde, ter dosado creatinina alguma vez, conhecer alguém com doença renal e maior idade. Na análise multivariada, a principal variável relacionada com conhecer a função da creatinina foi ter realizado o exame anteriormente (OR 5,16; IC 95% 3,16–8,43, p < 0,001).

**Conclusão:**

Há grande desconhecimento sobre a creatinina e seu uso em *check-ups*. Os resultados indicam que é necessário maior esforço por parte dos profissionais de saúde para divulgar o papel da creatinina sérica.

## Introduction

Chronic kidney disease (CKD) is a common condition that represents a public health issue nowadays. This becomes more evident when considering that the main causes of CKD are diabetes mellitus and hypertension, two diseases of high prevalence worldwide. CKD is defined as the presence of functional and/or structural changes in the kidneys for a period equal to or greater than three months^1,2^.

In a study conducted in the general population of the United States from 2013 to 2016, the estimated prevalence of CKD was approximately 15%. When stratified according to etiology, the prevalence of CKD in the group with diabetes mellitus (DM) was 36%, and in the group with hypertension, 31%^3^. In a systematic review from 2017, it was estimated that the prevalence of CKD in Brazil was around 3 to 6 million^4^. Meanwhile, the 2021 Dialysis Survey by the Brazilian Society of Nephrology (SBN) reported that, in July of the same year, the estimated total number of dialysis patients was 148,363. In addition, the estimates of prevalence and incidence rates of patients undergoing dialysis treatment per million of population were 696 and 224, respectively^5^.

As it is generally an asymptomatic or mildly symptomatic disease, the best means of preventing CKD is early diagnosis through laboratory tests, including the measurement of serum creatinine. This test is of considerable value for assessing kidney function, with the advantages of being simple and widely available^6^.

One of the important approaches taken by CKD prevention campaigns in Brazil and around the world is to increase the general population’s awareness of the disease and how to achieve early diagnosis^7^. Thus, having a picture of how much the population is aware of this biomarker, serum creatinine, which is crucial for diagnosing CKD in clinical practice, is important to assess whether the approaches used so far have yielded satisfactory results. It is also crucial for planning how to communicate this and other prevention information to people.

Since health education for the general population is associated with levels of quality of life and prevention, the aim of this study was to assess, in a sample of the Brazilian population, knowledge about the role of serum creatinine as a marker of kidney function.

## Methods

A cross-sectional observational study was conducted in São Paulo (SP, Brazil) in 2023, assessing a sample of the population regarding their knowledge on the role of serum creatinine as a biomarker of kidney function. Open Epi^8^ software was used to calculate the sample size, which was 1068 people, using a confidence level of 95%, a 3% margin of error and a population proportion of 50%, as there is no previous data available on the frequency of knowledge in the population.

Data were collected through individual interviews after the subjects signed the Informed Consent Form (ICF). Participants were randomly invited for interviews in public places, and the inclusion criteria were: being Brazilian and aged 18 or over.

All data collection was conducted on the same high-traffic avenue in São Paulo, Avenida Paulista, along its entire length. This avenue is in a central neighborhood, with commercial/business, residential (to a lesser extent) and tourist components, frequented by different social classes and age groups.

Due to the lack of a validated questionnaire for our purpose, a form was created with information considered relevant by the authors. The following aspects were considered: age, sex, level of education, whether or not they were a healthcare professional or student, whether or not they had previously undergone medical checkups and how often, previous testing for serum levels of cholesterol, glucose and creatinine, whether they knew the purpose of the creatinine test, whether or not the participant had been diagnosed with diabetes and/or hypertension, and whether they knew people with kidney disease.

After the interview, the participants were provided with information about the role of serum creatinine in assessing kidney function and, especially, as a diagnostic tool for CKD.

The study was approved by the Research Ethics Committee (UNIFESP), under protocol 5.721.239.

### Statistical Analysis

The IBM SPSS 25.0 software was used for tabulating and analyzing the results and the GraphPad Prism 8.0 program was used to create figures. For continuous variables, data were described as median and 1^st^ and 3^rd^ interquartile ranges, while categorical variables were presented as frequencies and percentages. Chi-square and the Mann-Whitney U-test for 2 independent samples with non-normal continuous variables were the statistical tests used to compare categorical variables. Multivariate analysis using binary logistic regression was performed to assess the influence of variables potentially associated to creatinine accuracy rate, after checking for collinearity between selected variables. *Odds ratio* (OR) and 95% confidence interval (95% CI) were calculated. A statistically significant difference was assumed when p < 0.05 in a two-tailed test.

## Results

**Figure 1.**
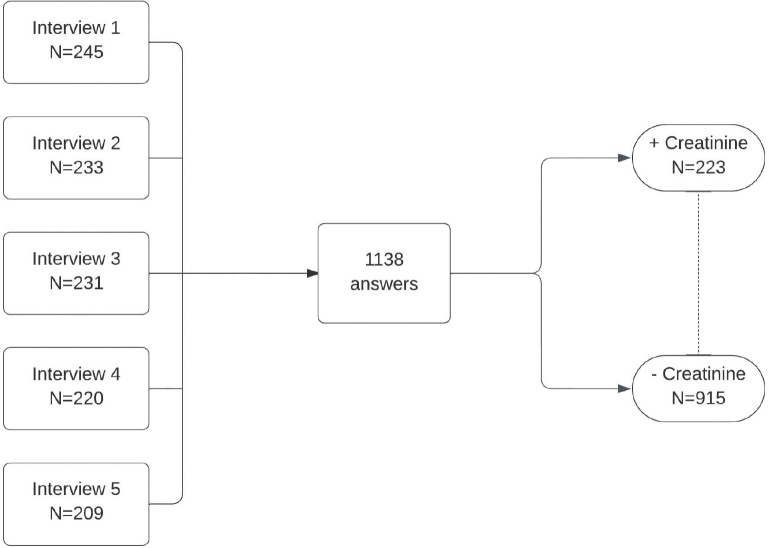
Flowchart of the study implementation, including the number of participants per interview day, total number of participants and interviewees who knew (+) or did not know (–) the role of serum creatinine.

**Figure 2.**
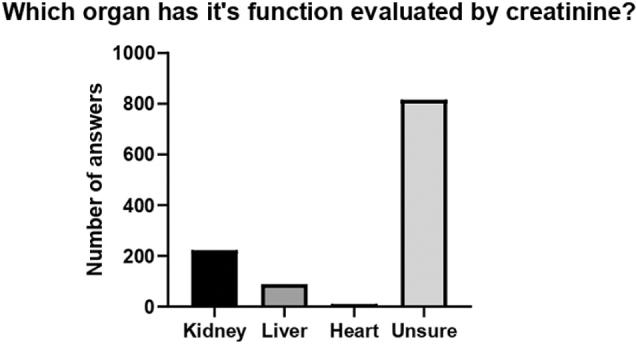
Responses (4 options provided) to the question: “Serum creatinine is used to assess the function of which organ?”

A total of 1138 people were interviewed on 5 different days. General data were analyzed, and two groups were compared (correctly identified creatinine role vs. incorrectly identified creatinine role) (Figure 1). Demographic data and information obtained from the interviews are summarized in Table 1. The median age of the participants was 36 years old (27–52), and the majority were female (55.1%). A small proportion reported having hypertension and diabetes, 11.9% and 5.5% respectively. When dividing education level into 4 groups (each group included both complete and incomplete levels of the respective education), it was found that most participants had higher education (48.6%). The prevalence of healthcare students and workers was 5.9% and 14.0%, respectively. Approximately 75% of participants reported undergoing checkups, with an annual frequency predominating (45.2%). Cholesterol, blood glucose and serum creatinine measurements were reported to have been performed at some point by 81.5%, 83.5% and 59.4% of respondents. Of those who reported having undergone a serum creatinine test, 90% answered that the test had a normal result. Regarding knowing people with kidney disease, 47.8% of participants reported knowing someone, either themselves (12.7%), family members (52.5%) or others (34.8%). Of the total number of participants, 223 (19.6%) answered correctly about the role of creatinine among the four options provided, while 817 (71.6%) answered “do not know”. The remaining chose the answers “assess liver function” (89; 7.9%) and “assess heart function” (10; 0.9%) (Figure 2).

**Table 1.** Demographic data and information collected through interviews

Variables	All	Correct*	Incorrect*	P
	n (%) / Median (1st – 3rd IQ)			
N	1138	223 (19.6)	915 (80.4)	
Age, years	36 (27–52)	41 (30–57)	36 (26–51)	*<0.001*
Sex				
Female	627 (55.1)	139 (62.3)	488 (53.3)	*0.015*
Male	511 (44.9)	84 (37.4)	427 (46.7)	
Comorbidities				
Hypertension	135 (11.9)	37 (16.6)	98 (10.7)	*0.015*
Diabetes	63 (5.5)	17 (7.6)	46 (5)	0.138
Education				
Elementary	59 (5.2)	5 (2.2)	54 (5.9)	*<0.001*
High School	321 (28.2)	35 (15.7)	286 (31.3)	
Higher Education	553 (48.6)	119 (53.4)	434 (47.4)	
Postgraduate	178 (18.0)	64 (28.7)	141 (15.4)	
Healthcare students	67 (5.9)	29 (12.0)	38 (4.2)	*<0.001*
Healthcare workers	159 (14.0)	76 (34.1)	83 (9.1)	*<0.001*
Checkup				
No	280 (24.6)	31 (13.9)	249 (27.2)	*<0.001*
Once a year	514 (45.2)	123 (55.2)	391 (42.7)	
Twice a year	255 (22.4)	55 (24.7)	200 (21.9)	
Less than once a year	89 (7.8)	14 (6.2)	75 (8.2)	
Previous test measurements				
Cholesterol	928 (81.5)	212 (95.1)	716 (78.3)	*<0.001*
Glucose	952 (83.7)	214 (96.0)	738 (80.7)	*<0.001*
Creatinine				
No	462 (40.6)	24 (10.8)	438 (47.9)	*<0.001*
Normal	609 (53.5)	185 (83.0)	424 (46.3)	
Abnormal	32 (2.8)	11 (4.9)	21 (2.3)	
Doesn’t remember	35 (3.1)	3 (1.3)	32 (3.5)	
Acquaintance with kidney disease**	544 (47.8)	145 (65.0)	399 (43.6)	*<0.001*

* The participant correctly or incorrectly identified the creatinine role during the interview;

** Participant knows someone with kidney disease.

When comparing the two groups (correct vs. incorrect), the “correct” group was older, had more female participants and a higher level of education, had undergone more checkups, cholesterol, glucose, and serum creatinine tests, more frequently knew someone with CKD, had a higher prevalence of hypertension and was a healthcare student/worker. In the healthcare workers group, the physicians answered correctly in 91.4% of cases. Among those who reported having previously tested for serum creatinine, 29.4% answered correctly about the function of this laboratory test.

**Figure 3.**
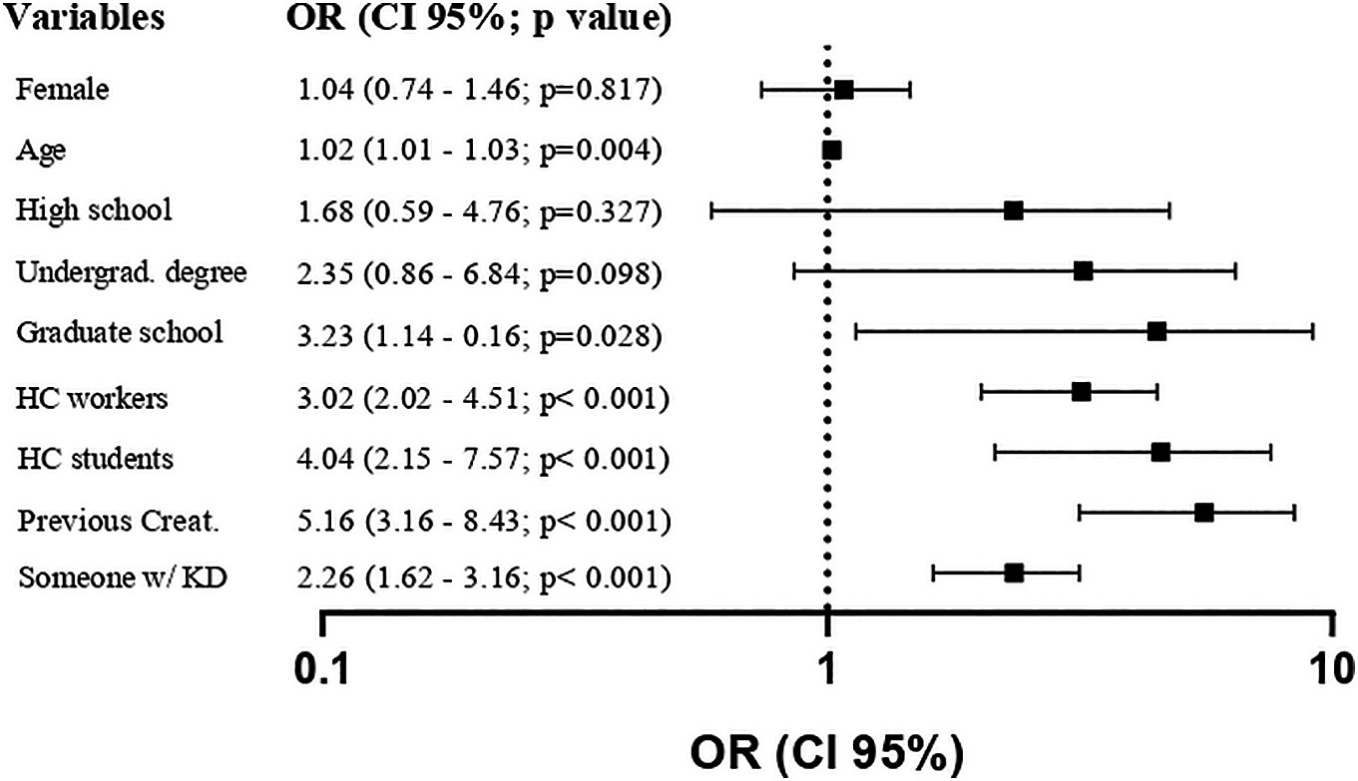
Forest plot relating different variables to knowledge on serum creatinine role using multivariate logistic regression. Abbreviations: H. School = High School; H. Education = Higher Education; HC = Healthcare; Creat. measurement = Previous serum creatinine measurement; Know KD = Know someone with kidney disease.

In the multivariate analysis, the main potentially related variables (Figure 3) – being older, having a higher level of education (postgraduate), being a healthcare student/worker, having previously undergone a creatinine test and knowing someone with kidney disease – were positively associated with answering correctly about the role of the “creatinine” biomarker.

## Discussion

One way of preventing CKD is to educate the population, healthcare professionals and policymakers about the disease, which has been a public health problem for decades^7^. It is known that the complexity of informing the population is significant, so we sought to identify the extent to which the general population, interviewed without prior selection in public places in a Brazilian city, is aware of serum creatinine function. This biomarker was chosen for its relevance in assessing kidney function and its universal applicability as a diagnostic tool for CKD.

It should be noted that, according to data from the NHS^9^ (National Health Survey), chronic diseases experienced a generalized increase from 2013 to 2019. During the period when the NHS was conducted, the percentage of the population reporting at least one chronic disease increased from 45% to 50.8% (an increase of 12.8%). Among the chronic noncommunicable diseases studied, DM increased the most, from 6.2% of the population in 2013 to 7.7% in 2019 (a 24% increase). Another notable disease in the analysis of NHS data was hypertension, with an increase of 12% (21.4% in 2013 and 23.9% in 2019). CKD was also subject of study, experiencing a 7% increase nationwide. It is worth noting that the increase in DM and hypertension is also associated with a higher population risk of developing CKD.

In this study, the population sample interviewed was comprised of adults, with a median age of 36 years and a slight predominance of females, which is in line with the current distribution of the total Brazilian population. It is worth noting that, according to the 2022 survey, 48.9% of Brazilians were male and 51.1% were female. Among people aged up to 24, men are the majority. In the 25–29 age group, the proportion of men and women is similar. From the age of 30 onwards, women outnumber men, and this was the predominant age group in the present study. Above 60 years old, this difference increases even further^10^.

With regards to education, we observed a predominance of individuals with a high level of education among our interviewees. This is in line with the recent finding of an increase in the average level of education in the country from 2016 to 2022, according to the Continuous National Household Sample Survey by IBGE^10^. When evaluating education according to the age distribution of the Brazilian population, 53.2% of individuals aged 25 or older had completed compulsory basic education, meaning they had completed at least high school. In addition, 19.2% of individuals in this age group had completed higher education^10^.

Education is known to be one of the key factors associated with health literacy, alongside income and socioeconomic background. It is worth remembering that health literacy refers to cognitive and social skills, which determine the motivation and ability of an individual to understand and use information to promote good health, prevent diseases and improve quality of life^11^.

Previous studies assessing knowledge about chronic diseases such as CKD^11^, DM^12^ and hypertension ^13^, among others, have shown that lower educational levels are generally related to poorer understanding of diseases, prevention and treatment factors, and lower health literacy.

In a previous Brazilian study involving a questionnaire administered to the general population regarding knowledge of CKD and serum creatinine, a higher level of education was associated with better knowledge. Participants with a higher education level had more correct answers, while illiterate individuals or those with elementary education had fewer correct answers. In the same study, younger adults demonstrated more correct answers to questions about risk factors and prevention methods for CKD^11^. In another study, this time involving patients with kidney diseases, low education and older age were predictive factors for lower functional health literacy^14^.

One of the topics covered in our questionnaire was the performance of checkups, which are referred to by several different terms, such as general health exams, general medical exams, periodic health exams, wellness visits and others. These are often used for diagnosis and prevention of diseases and are commonly performed in primary care, being associated with increased identification and treatment of chronic diseases^15^.

In the present study, we tried to identify how often participants underwent checkups, noting a high frequency of prior blood glucose and serum cholesterol tests at the time of their checkups, accounting for 83.7% and 81.5%, respectively. In Brazil as a whole, available data indicate that percentages of the population slightly lower than these usually go for checkups. According to the IBGE Census conducted in 2021, around 33% of the Brazilian population did not undergo annual checkup exams and, in 2018, based on a study by the Ministry of Health, one out of three Brazilians did not attend medical appointments on a regular basis.

Serum creatinine, on the other hand, was mentioned as part of checkups in less than 60% of cases. As far as blood glucose and cholesterol tests are concerned, we couldn’t find a similar previous publication, but our results are at least proportional to data from the NHS^9^, released in 2019, which reported that the percentage of Brazilians aged 18 or over who had never been tested for blood glucose and serum cholesterol was approximately 6.2% (5.9-6.6%) and 7.5% (7.2-7.9%), respectively.

The authors acknowledge the limitations of the current study, especially the potential lack of representativeness of the sample, although an adequate sample size was included, highlighting the fact that the interviews were conducted in only one city in the country. Such limitations may impact the results and their generalization, since this sample of the population does not entirely represent the composition of the Brazilian population, including in terms of educational level. A greater proportion of individuals with a high level of education could contribute to an overestimation of the true percentage of the population familiar with the function of serum creatinine.

Nevertheless, our results indicate that the population’s level of knowledge on the subject is still low. This information could certainly be considered in the planning of preventive actions by healthcare professionals involved in caring for patients with CKD and by those responsible for health policymaking, especially when conducting prevention campaigns, the results of which are challenging to quantify.

At this point, it is worth recalling some of the strategies to enhance the awareness of the population on CKD and its prevention. This includes describing the basic laboratory markers for early diagnosis, such as serum creatinine and urine tests (especially proteinuria screening), as discussed by Mastroianni-Kirsztajn et al.^7^ Some of these strategies include the production and distribution of educational material adapted to each location (leaflets, booklets, posters, etc.) presented in simple and illustrated language to reach individuals at all levels of education. Campaigns have been organized to raise visibility on these issues, particularly the celebration of World Kidney Day. These campaigns involve a wide range of events, interviews in different media, lighting up monuments, celebrations, joint efforts to provide care and screening for CKD, billboard advertising, and other resources.

In summary, when evaluating a random sample of the Brazilian population, approached in public spaces in the city of São Paulo, we observed a 75% rate of undergoing checkups, at least on an annual basis, in line with the data published by the IBGE. In the tests conducted by the interviewees, the frequency of creatinine measurement was lower than that of other laboratory tests, such as glucose and cholesterol, which are crucial in screening chronic noncommunicable diseases; in the case of creatinine, around 60%. What really drew our attention was the low awareness (20% of correct answers) about the functional role of serum creatinine, even though there were few response options provided. We believe that increasing awareness about the role of this biomarker could contribute to its more frequent inclusion in checkups and tests performed by different specialists. This might become a demand from individuals themselves, who have come to know the importance of this test in the early diagnosis of CKD. We also emphasize that, to make population education possible and consistent, health professionals and services, including those not specialized in Nephrology, should be aware of and prepared to continue the process of education and diagnosis of CKD as well. For us, the importance of promoting the need to measure serum creatinine as a means of early diagnosis of CKD in any checkup has become clear, especially when evaluating patients who are part of risk groups for developing CKD.

Finally, we emphasize that in the current study, after the questionnaires were administered, the interviewees received information on the role of serum creatinine in the diagnosis and prevention of CKD.
